# Potassium in CKD: Friend or Foe?

**DOI:** 10.34067/KID.0000000658

**Published:** 2024-12-26

**Authors:** Sharon Turban, Paola Gudino, Michal L. Melamed

**Affiliations:** 1 Division of Nephrology, Johns Hopkins School of Medicine, Baltimore, Maryland; 2 Department of Medicine, Jacobi Medical Center/Albert Einstein College of Medicine, Bronx, New York; 3 Department of Medicine, NYU Grossman School of Medicine, New York, New York

**Keywords:** potassium (K) channels, progression of renal failure

## Introduction

The relationship between elevated potassium (K^+^) levels and health outcomes remains a key issue for individuals with CKD. The role of K^+^ in CKD is not fully understood, particularly regarding the long-term effect of dietary potassium intake and serum K^+^ levels on kidney disease progression and cardiovascular (CV) outcomes. Managing K^+^ in this population can be complex because hyperkalemia, which can be life threatening, becomes more frequent as CKD progresses. Hyperkalemia is also more prevalent in individuals on renin-angiotensin-aldosterone system (RAAS) inhibitors, which are frequently prescribed to individuals with CKD for their protective effects on CV and kidney health. However, overly restrictive dietary potassium limitations can compromise nutritional quality and reduce potential CV and kidney effects of a potassium-rich diet. Diets rich in K^+^, particularly those high in fruits and vegetables, may provide CV and kidney benefits, including reducing BP and potentially slowing kidney disease progression. It is important to differentiate between dietary potassium intake and serum K^+^ levels. Serum K^+^ levels are affected by both intake and excretion, with impaired K^+^ excretion in CKD. In addition, serum K^+^ levels may be affected by shifts from the intracellular to extracellular space, as may occur in the setting of diabetes mellitus or metabolic acidosis.

In this issue of *Kidney360*, Agiro *et al.* present results from an analysis of a large health care database study where the association between hyperkalemia and outcomes using propensity score–matched cohorts was evaluated. The authors found that individuals with stage 3b/4 CKD, not prescribed regular outpatient intestinal K^+^ binders, who experienced at least one episode of hyperkalemia (defined as serum K^+^ >5.0 mmol/L) had a 1.60-fold (95% confidence interval [CI], 1.50 to 1.71, *P* < 0.001) higher risk of CKD progression and 1.09-fold (95% CI, 1.02 to 1.16; *P* < 0.01) higher risk of all-cause mortality relative to individuals without hyperkalemia.^1^ This study provides important new data, although interpreting the results requires caution because of multiple reasons outlined in this commentary.

## Association of Hyperkalemia with Adverse Outcomes

Associations of hyperkalemia with morbidity and mortality have previously been reported. Hyperkalemia has been associated with increased CV mortality, potentially due to the heightened risk of arrhythmias. An analysis of almost 250,000 veterans showed that an episode of hyperkalemia, defined as a K^+^ >5.5 mmol/L was associated with a higher risk of mortality within 24 hours.^2^ A large, multinational meta-analysis demonstrated that serum K^+^ levels >5.5 mmol/L were associated with higher risks of progression to kidney failure and all-cause mortality compared with a serum K^+^ concentration of 4.2 mmol/L.^3^ However, the findings also demonstrated that the adjusted hazard ratio of all-cause mortality was higher for *hypo*kalemia (hazard ratio: 1.49 [95% CI, 1.26 to 1.76] at 3.0 mmol/L).^3^ Thus, both hyper- and hypokalemia are associated with mortality.

This study by Agiro *et al.* builds on these previous studies and adds a rigorous study design with propensity score matching to try to overcome some of the limitations of observational studies. However, it remains unclear whether hyperkalemia directly causes poor outcomes or merely reflects underlying disease severity. The higher risk seen in the study may also be driven by medication changes in response to hyperkalemia, such as the discontinuation of RAAS inhibitors. Reverse-causation bias is a concern because stopping RAAS inhibitors because of hyperkalemia may contribute to the observed risk. Evidence shows that RAAS inhibition slows CKD progression and improves CV outcomes, but hyperkalemia often leads to the downtitration or discontinuation of these therapies. Leon *et al.* demonstrated that discontinuing RAAS inhibitors was associated with a higher mortality and CV disease events compared with continuation in CKD patients with hyperkalemia.^4^ Another factor to consider in this setting is that patients may be advised to reduce dietary K intake excessively after a hyperkalemic episode, depriving them of the CV and kidney benefits of a plant-based diet.

## Beneficial Effects of Potassium-Rich Diets

Although hyperkalemia has been linked to negative outcomes, it is important to consider the benefits of dietary potassium. Potassium-rich foods, such as fruits and vegetables, offer CV benefits that are independent of serum K^+^ levels. With CKD increasing the risk of CV disease and mortality, evidence suggests that a plant-based, potassium-rich diet can reduce BP, improve CV health, and potentially slow kidney disease progression. In addition, such a diet may prevent constipation and mitigate metabolic acidosis, which can reduce hyperkalemia risk.

Data from animal studies, human epidemiological research, and limited clinical intervention trials highlight the benefits of potassium-rich diets in CKD. A systematic review of mostly observational studies found that higher urine K^+^ was associated with slower CKD progression and improved outcomes in predialysis patients.^5^ Another study of 240 patients with CKD stages 3–5 demonstrated that higher 24-hour urinary K^+^ excretion correlated with slower declines in eGFR, underscoring the potential advantages of maintaining potassium-rich diets even in advanced CKD.^6^

## Study Design and Potential Confounders

This observational study by Agiro *et al.* uses propensity score matching to evaluate the relationship between hyperkalemia and CKD outcomes.^1^ Although this method helps address some confounding, residual confounders remain a challenge; the observational nature of the study still poses inherent limitations, particularly concerning the establishment of causality. The study does not account for several pertinent potential confounders and mediators, such as metabolic acidosis, episodes of hypokalemia, changes in RAAS inhibitor dosing, diet composition, proteinuria quantification, and use of nonsteroidal anti-inflammatory drugs or other medications that may affect K^+^ concentration. When analyzing the underlying cause of CKD progression, these variables need to be carefully considered. For example, metabolic acidosis and hyperkalemia often exist together and both separately are associated with progression of kidney disease. While there is potential biologic rationale for either causing CKD progression, they may also simply be markers for worse GFR not captured in a creatinine-based eGFR calculation. The complexity of establishing clear causality arises from the fact that the elevated K^+^ levels may be a consequence of or marker of disease progression rather than the cause.

## The Challenge of Isolated Hyperkalemic Events

The influence of a single hyperkalemic event on long-term outcomes remains insufficiently evaluated. Inclusion for the hyperkalemia group in the study by Agiro *et al.* required a single serum K^+^ >5.0 mmol/L, which is lower than what some guidelines define as hyperkalemia.^1^ However, the implications of one episode of hyperkalemia are different from chronic hyperkalemia. Furthermore, isolated episodes of elevated serum K^+^ may be related to transient events such as superimposed episodes of AKI, transient use of medications (*e.g*., trimethoprim, nonsteroidal anti-inflammatory drugs, K-sparing diuretic) or even pseudohyperkalemia.^7^ In the study by Agiro *et al.*, there was no requirement for corroboration by whole-blood K^+^ or another serum K^+^ value.

## Possible Mechanisms for Kidney Disease Progression after a Hyperkalemic Episode

As previously stated, it may be that hyperkalemia is just a marker of already worse kidney disease unmeasured by creatinine-based eGFR. Two potential mechanisms for the observed findings are physicians stopping the RAAS inhibitors or recommending a low-potassium diet after hyperkalemia. Both of these are commonly done in clinical practice. In this commentary, we argue that these may not be the best course of action after an episode of hyperkalemia. Alternatively, using a K^+^ binder, such as patiromer, can help patients continue on their RAAS inhibitors. A recent study demonstrated that long-term patiromer use in CKD patients with hyperkalemia was associated with a lower risk of all-cause mortality compared with those not taking patiromer.^8^ Importantly, although there is an association between hyperkalemia and kidney disease progression found in the study by Agiro *et al.*, the effect of lowering serum K^+^ levels (*e.g*., by dietary change or potassium binder) has not been well studied.

## Conclusion

While the study by Agiro *et al.* provides valuable insights into the association between hyperkalemia, CKD progression, and mortality, it also underscores the complexity of potassium management in this population. Elevated serum K^+^ may reflect more advanced underlying kidney disease rather than serve as a direct cause of poor outcomes. Confounding factors, such as declining kidney function, medication effects, and acute illnesses, must be accounted for when interpreting these findings. Prematurely restricting potassium-rich diets or discontinuing RAAS inhibitors could deprive patients of critical CV and kidney benefits, ultimately potentially doing more harm than good. The goal of clinical care should be to attempt to optimize potassium management without compromising nutrition or essential therapies. Potassium binders offer a practical strategy to help achieve this balance. A nuanced, individualized approach that weighs both the benefits and risks is essential to improving outcomes in patients with CKD. Well-designed clinical trials are needed to better understand the long-term effects of potassium intake and serum K^+^ levels on kidney disease progression and CV health, as well as how to best prevent and manage hyperkalemia.
